# Broadly sampled multigene trees of eukaryotes

**DOI:** 10.1186/1471-2148-8-14

**Published:** 2008-01-18

**Authors:** Hwan Su Yoon, Jessica Grant, Yonas I Tekle, Min Wu, Benjamin C Chaon, Jeffrey C Cole, John M Logsdon, David J Patterson, Debashish Bhattacharya, Laura A Katz

**Affiliations:** 1 Department of Biological Sciences and Center for Comparative Genomics, University of Iowa, Iowa City, Iowa 52242, USA; 2 Department of Biological Sciences, Smith College, Northampton, MA 01063, USA; 3 American Type Culture Collection, P.O. Box 1549, Manassas, VA 20108, USA; 4 Marine Biological Laboratory, Woods Hole, MA 02543, USA; 5 Bigelow Laboratory for Ocean Sciences, 180 West Boothbay Harbor, ME 04575, USA

## Abstract

**Background:**

Our understanding of the eukaryotic tree of life and the tremendous diversity of microbial eukaryotes is in flux as additional genes and diverse taxa are sampled for molecular analyses. Despite instability in many analyses, there is an increasing trend to classify eukaryotic diversity into six major supergroups: the 'Amoebozoa', 'Chromalveolata', 'Excavata', 'Opisthokonta', 'Plantae', and 'Rhizaria'. Previous molecular analyses have often suffered from either a broad taxon sampling using only single-gene data or have used multigene data with a limited sample of taxa. This study has two major aims: (1) to place taxa represented by 72 sequences, 61 of which have not been characterized previously, onto a well-sampled multigene genealogy, and (2) to evaluate the support for the six putative supergroups using two taxon-rich data sets and a variety of phylogenetic approaches.

**Results:**

The inferred trees reveal strong support for many clades that also have defining ultrastructural or molecular characters. In contrast, we find limited to no support for most of the putative supergroups as only the 'Opisthokonta' receive strong support in our analyses. The supergroup 'Amoebozoa' has only moderate support, whereas the 'Chromalveolata', 'Excavata', 'Plantae', and 'Rhizaria' receive very limited or no support.

**Conclusion:**

Our analytical approach substantiates the power of increased taxon sampling in placing diverse eukaryotic lineages within well-supported clades. At the same time, this study indicates that the six supergroup hypothesis of higher-level eukaryotic classification is likely premature. The use of a taxon-rich data set with 105 lineages, which still includes only a small fraction of the diversity of microbial eukaryotes, fails to resolve deeper phylogenetic relationships and reveals no support for four of the six proposed supergroups. Our analyses provide a point of departure for future taxon- and gene-rich analyses of the eukaryotic tree of life, which will be critical for resolving their phylogenetic interrelationships.

## Background

A major remaining gap in our knowledge of the tree of life is the uncertain relationships among eukaryotes, including the many divergent microbial lineages plus plants, animals and fungi. Microbial eukaryotes, often referred to as protists, are an eclectic assemblage of lineages that are defined as eukaryotes that are not plants, animals, or fungi [1]. Clearly, knowledge of the phylogenetic positions of protists is a key to understanding the origins of eukaryotes, and where the ancestries of plants, animals and fungi lie within these microbial groups.

During the 1970's and 1980's a revolution in understanding eukaryotic diversity occurred as a result of ultrastructural studies. These data [2,3] demolished traditional classifications where algae, protozoa and fungi were considered discrete entities, and microbial eukaryotes were inappropriately lumped into one of four classes: amoebae, flagellates, ciliates, and sporozoans. Ultrastructural studies revealed distinct assemblages of organisms that are distinguished by their complement and organization of organelles, providing lineages with ultrastructural identities [1]. About 60 different, robust patterns of ultrastructural organization are recognized, but ~200 genera of uncertain affinities have yet to be examined [1,4]. Determining relationships among groups using ultrastructure, however, has proven difficult, largely due to the lack of unambiguously homologous structures.

Early molecular analyses relied on comparisons of rDNAs from diverse protists and suggested that diplomonads, trichomonads, and microsporidia were basal lineages [5-7]. These analyses of rDNAs sequences also produced a topology with a base and crown (putatively recently radiated) lineages [8-11], which is now argued to be an artifact of long branch attraction. Given the well known limitations of single gene genealogies when inferring deep evolutionary relationships, the current trend is to focus on multigene datasets [12,13]. However, taxon representation in many of these analyses is sparse. With such incomplete taxon sampling, distantly related groups may appear as sister taxa and many deep nodes are poorly supported [14].

The past decade has seen the emergence of six eukaryotic 'supergroups' that aim to portray evolutionary relationships between microbial and macrobial lineages. The supergroup concept is increasingly accepted as evidenced by several reviews [15,16] and the recently proposed formal reclassification by the International Society of Protozoologists [17]. However, the support for supergroups is highly variable in the published literature [14].

The six putative supergroups have complex and often unstable histories. The supergroup 'Amoebozoa' was proposed in 1996 [18,19] based largely on molecular genealogies. The controversial supergroup 'Chromalveolata' was proposed based on the assertion that the last common ancestor of the 'Chromista' (cryptophytes, haptophytes, stramenopiles) and the undisputed Alveolata (dinoflagellates, apicomplexans, ciliates) contained a common chlorophyll *c*-containing red algal plastid [20]. 'Excavata' is another controversial supergroup composed predominately of heterotrophic flagellates whose ancestor is postulated to have had a synapomorphy of a conserved ventral feeding groove [21]. The supergroup 'Opisthokonta' includes animals, fungi, and their microbial relatives, and is supported by many molecular genealogies [10]. The 'Opisthokonta' is united by the presence of a single posterior flagellum in many constituent lineages [22]. The supergroup 'Plantae' was erected as a Kingdom in 1981 [23] to unite the three lineages with double-membrane primary plastids: green algae (including land plants), rhodophytes, and glaucophytes. Finally, the 'Rhizaria' emerged from molecular data in 2002 to unite a heterogeneous group of flagellates and amoebae including: cercomonads, foraminifera, some of the diverse testate amoebae, and former members of the polyphyletic radiolaria [24].

We believe that comprehensive taxon sampling, coupled with gene-rich analyses, is critical for resolving accurate phylogenies [14]. This is particularly relevant for the eukaryotes where only a tiny fraction of the >200,000 species of microbial eukaryotes have thus far been characterized for any gene sequence, and over one-half of identified protists groups [1] have yet to be subjected to any molecular study. Misleading results can also arise if a study addressing "deeper" splits in the eukaryotic tree does not include a broad diversity of lineages, including members of all six putative supergroups [14]. This is because the addition of diverse lineages is critical to break long single branches that pose a significant problem for robust phylogenetic inference. We know that the lack of adequate sampling and the use of highly derived (e.g., parasitic) taxa have created unstable tree topologies and led to inaccurate statements of sister-group relationships (i.e., in the creation of the now-abandoned supergroup Archezoa, whose history is described in [25]). Yet only a handful of studies have been published that take a multigene taxon-rich approach for assessing the eukaryotic tree of life.

Here, we set out to accomplish two tasks: (1) place newly determined sequences from a diversity of microbial eukaryotes onto relatively well-sampled multigene eukaryote phylogenies, and (2) evaluate the support for the six supergroups. Our approach was to use phylogenetic analyses of four genes from two distinct taxon sets that included 61 newly-characterized sequences. The two taxon sets represent 1) 105 diverse eukaryotic lineages and 2) a reduced 92 taxon set in which long-branch taxa were removed. The four loci, SSU-rDNA, actin, alpha-tubulin, and beta-tubulin, have a rich history in eukaryotic phylogenetics [7,12,26]. These genes have been used for more intensive studies of some groups such as 'Amoebozoa' [27], 'Rhizaria' [28] and 'Opisthokonta' [29] as well as for the establishment of many of the proposed supergroups [14]. Yet, there are few studies in which a multigene data set has been combined with extensive taxon sampling from all six supergroups [30,31].

Our work contrasts with many past efforts that have used either single-gene data with a broad taxon sampling [8-11], or multigene data with a limited number of taxa [12,13,26,32]. We performed individual and concatenated analyses of four genes. To assess rate heterogeneity and possible lateral gene transfers, we analyzed each gene individually prior to concatenation and then applied a variety of phylogenetic inference methods with both DNA and the inferred protein sequences. Use of a concatenated data set greatly reduces phylogenetic error in simulation studies [33] and the large number of characters that we have obtained for this study is expected to improve the accuracy of resulting phylogenetic trees [34].

Seventy-two sequences were characterized for this study, the bulk of which are newly-characterized (47 sequences) or were previously characterized from other strains (14 sequences), were available as ESTs in public databases (1 sequence) or are previously published and confirmed here (10 sequences; see Additional file 1). These sequences include representatives of all six 'Chromalveolata' groups thereby sampling a sizable fraction of the diversity in this supergroup. This is critical with respect to overall eukaryotic diversity because 'Chromalveolata' contain about one-half of the recognized species of protists and algae [35]. In addition, eight of the ten 'Excavata' lineages were included in our study. Finally, we also add genes from several lineages within the 'Rhizaria', another poorly supported eukaryotic supergroup [14].

## Results and Discussion

### Sampling strategy

The 105-taxon dataset was chosen because the included taxa: (1) contained at least three of the four targeted genes; and (2) represented the known breadth of eukaryotic diversity (see additional file 1). These 105 diverse lineages represent 26 well-established eukaryotic groups as well as those of uncertain affiliation (e.g., *Ancyromonas*, *Malawimonas, Stephanopogon*), and contained members of all six putative supergroups (see Additional file 1). As discussed below, a second data set containing 92 taxa was generated by removing known problematic taxa including long branched ciliates [36], foraminifera [37,38], *Giardia *[39], plus several others (see Additional file 1).

**Figure 1:**
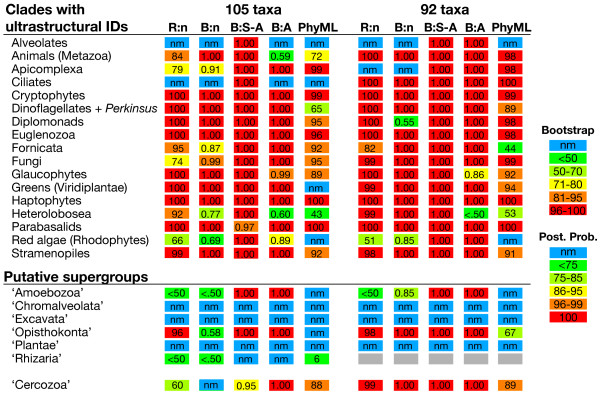
**Overall evaluation of support for nodes in 105 and 92 taxon sets**. Support for nodes using different taxon datasets, algorithms and evolutionary models. The top portion lists taxa with clear ultrastructural identities [1] whereas taxa below the line represent putative eukaryotic supergroups. As described in the text, two datasets were examined (105 and 92 taxa). **R:n **= RAxML analysis of 4 genes as nucleotides, with third codon positions removed; **B:n **= Bayesian analysis of 4 genes as nucleotides, with third codon positions removed; **B:S-A **= Bayesian analysis of the SSU-rDNA gene and the remaining three genes as amino acids; **B:A **= Bayesian analysis of three genes as amino acids; **PhyML **= Likelihood analysis under PHYML of three genes as amino acids. The 'Rhizaria' are indicated by grey boxes for the 92 taxon analysis as there were insufficient taxa to address this hypothesis; instead, we report support for the nested group 'Cercozoa.'

**Figure 2:**
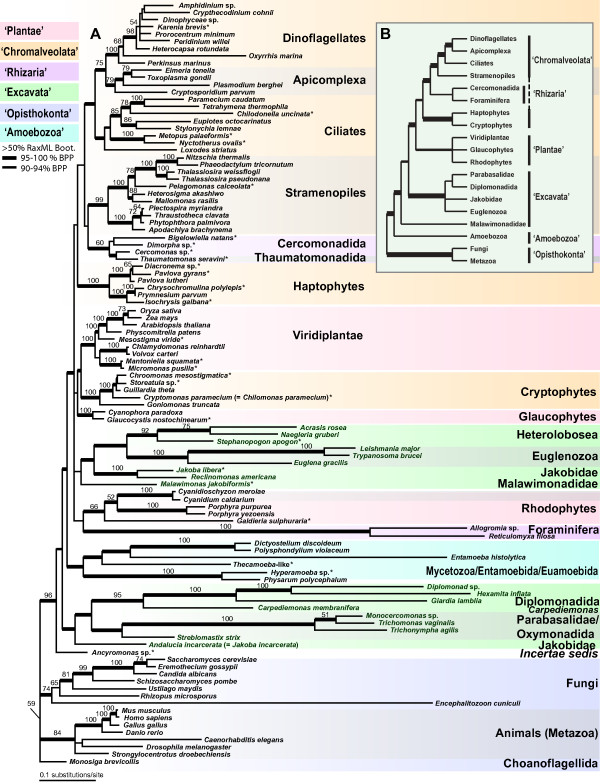
**Eukaryotic phylogeny based on 105 taxon set**. (A) Phylogeny of the major eukaryotic groups inferred from a Bayesian analysis of the combined SSU-rDNA and amino acid sequences of actin, alpha-tubulin, and beta-tubulin from 105 taxa. This is the 50% majority rule consensus tree with average branch lengths that were calculated from the Bayesian posterior tree distribution. Results of a RAxML bootstrap analysis are shown above the branches. Node thickness indicates >95% Bayesian posterior probability support, as indicated in the key. The branch lengths are proportional to the number of substitutions per site. The six eukaryotic supergroups are shown in different colors (see key). (B) Schematic phylogeny summarizing the results of Hackett et al. 2007. The thickest branches define clades that received ≥ 90% maximum likelihood bootstrap support, whereas the relatively thinner branches provide 80 – 89% bootstrap support. Stars indicate taxa that were characterized in this study (see additional file 1 for details).

**Figure 3:**
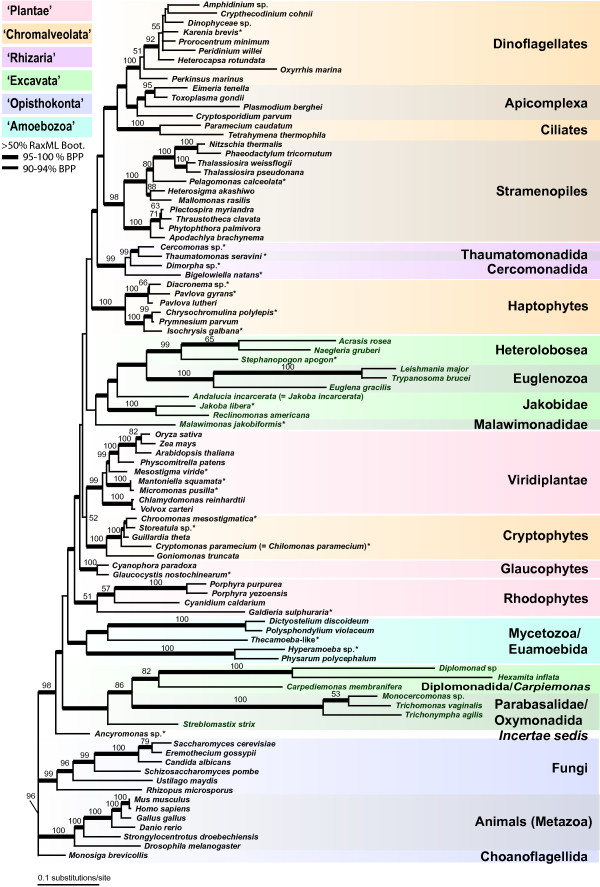
**Eukaryotic phylogeny based on 92 taxon set**. Phylogeny of the major eukaryotic groups inferred from a Bayesian analysis of the combined SSU-rDNA and amino acid sequences of actin, alpha-tubulin, and beta-tubulin from 92 taxa. Other notes as in Figure 2.

To assess the impact of evolutionary models and phylogenetic methods, both data sets (105 and 92 taxa) were analyzed under five combinations of data and methods: (1) a four-gene data set (SSU-rDNA, actin, alpha-, and beta-tubulin) as nucleotides excluding third codon positions using RAxML, (2) a four-gene data set as nucleotides excluding third codon positions using MrBayes, (3) a data set of mixed nucleotide (SSU) and amino acid sequences using MrBayes, (4) a three-gene data set (actin, alpha-, and beta-tubulin) as amino acids using MrBayes, and (5) a three-gene data set (actin, alpha-, and beta-tubulin) as amino acids using PHYML (Figs. 1, 2, 3 and see Additional files 2, 3, 4, 5, 6, 7, 8, 9). We also analyzed individual gene data sets to identify taxa that are characterized by high sequence divergence (i.e., a long-branch) or had an unexpected position in the phylogeny (see Additional files 10, 11, 12). Overall, there is some heterogeneity in support for hypotheses among models and algorithms but the major trends discussed below were consistent across the different analyses (summarized in Fig. 1).

### Placement of newly-determined sequences

We determined the position of newly characterized taxa using both the 105 (Fig. 2) and 92 (Fig. 3) taxon data sets. To simplify comparisons, all of the genealogies are drawn rooted with 'Opisthokonta', as hypothesized by Arisue *et al*. [40] and are generally concordant with previous studies using a similar set of genes [12,41]. We sampled additional genes from several members of the putative 'Rhizaria' including both the SSU rRNA gene and protein coding genes for a cercomonad *Cercomonas *sp. (ATCC PRA-21) and the dimorphid *Dimorpha *sp. (ATCC PRA-54), and protein coding genes for the thaumatomonad *Thaumatomonas seravini *(ATCC 50636; see Additional file 1). Relationships among these taxa generally agree with the proposed phylogeny of core 'Cercozoa' [42,43].

All four genes were analyzed for two amoeboid taxa – the myxogastrid *Hyperamoeba *sp. (ATCC PRA-39) and a *Thecamoeba*-like lineage (ATCC PRA-35) that is currently being described (Nerad *et al*., personal communication). *Hyperamoeba *sp. is sister to *Physarum *in all analyses except in the beta-tubulin protein tree (see Additional file 12). The *Physarum *+ *Hyperamoeba *sister group relationship was predicted on the basis of morphology [44]. The *Thecamoeba*-like lineage falls close to the mycetozoa plus Entamoebida. In contrast to published hypotheses [12,45], we do not find support for the monophyly of dictyostelids plus myxogastrids with our taxon and gene sampling. This finding is further supported by a well-sampled multigene analysis that included several previously-uncharacterized 'Amoebozoa' taxa [46].

We also included additional genes from several ciliates (*Nyctotherus ovalis, Metopus palaeformis *and *Chilodonella uncinata*). In many of the analyses, either there is a spurious relationship between ciliates and the Heterolobosea (e.g., see Additional files 2 and 3) or the ciliates are polyphyletic (see Additional files 4 and 5). As has been shown in previous work, ciliates are marked by considerable heterogeneity in protein evolution as rates of evolution are highly variable among lineages [36,47] and it is perhaps not surprising that increasing taxon sampling in this group fails to produce more stable trees. Use of the rDNA plus amino acid data for both 105 and 92 taxon data sets however recovers trees that support the expected monophyly of each of the lineages, ciliates, alveolates, and Heterolobosea (Figs. 1, 2, 3).

Furthermore, we characterized additional loci from two enigmatic taxa, the flagellate *Ancyromonas sigmoides *(ATCC 50267; species identity from [48]) and *Stephanopogon apogon *(ATCC 50096). In all our analyses, *S. apogon *shows a sister group relationship to the Heterolobosea (Figs. 1, 2, 3). *Stephanopogon apogon *has been suggested to be sister to the Euglenozoa based on mitochondrial cristae morphology and similarity in nuclear division profiles [49,50]. *Ancyromonas sigmoides *is a small, heterotrophic gliding flagellate with one recurrent and one anterior flagellum and flat mitochondrial cristae [1]. The phylogenetic position of *A. sigmoides *varies depending on which phylogenetic method is used. This is the case in other analyses of a subset of genes [14], suggesting that either there is inadequate sampling of this lineage or too few data to resolve its position, or that *Ancyromonas *represents a novel lineage of eukaryotes [51].

We also sampled representatives of all six chromalveolate phyla including multiple members from each phylum. Together, our analysis included new data for 12 taxa out of 43 chromalveolates in the tree. These 12 taxa were placed in their expected positions among the 6 different 'Chromalveolata' phyla, and all received moderate to strong support (i.e., apicomplexans, cryptophytes, haptophytes, dinoflagellates, stramenopiles) except for ciliates (see Figs. 1, 2). Within the 'Plantae' the 4-gene data support strongly the early divergence of *Mesostigma viride *within the streptophyte branch of the Viridiplantae. This is consistent with a recent multigene analysis of nuclear, chloroplast, and mitochondrial data; the 'Plantae' phyla Glaucophyta and Viridiplantae received strong bootstrap and Bayesian support whereas there was moderate support for Rhodophyta monophyly [52].

### Evaluation of supergroups

Putative eukaryotic supergroups receive mixed support in our analyses of the 105-taxon dataset. To improve our understanding of supergroup support, we compare these results with a recent studies including one [30] that used a concatenated alignment of 16-proteins but fewer (46) taxa (see inset in Fig. 1). In the present analyses, the supergroup 'Opisthokonta', receives high bootstrap support (>95%) and a significant posterior probability (1.00) under several of the different models and algorithms (R:n, B:S-A, B-A; Fig. 1). The relationship of at least some members of this supergroup emerged in previously published rDNA [10] and multigene analyses [12,30,41]. In addition, there are two compelling synapomorphies for this group: (1) the presence of a single flagellum in flagellate members of this group, with the flagellum 'posterior' in that it beats from base to tip and projects behind swimming cells, and (2) a unique amino acid insertion in those members that contain a canonical EF-1α gene [53] (some members of the 'Opisthokonta' have an EF-like protein and not EF-1α [54]). The inclusion of animals and fungi within 'Opisthokonta' refutes the monophyly of animals plus plants that has been suggested in some recent studies [55].

The putative supergroup 'Amoebozoa' receives high support only under a limited number of models and algorithms, including strong support (posterior probability = 1.00) under Bayesian analyses of amino acid sequences (B:A) and of SSU-rDNA as nucleotides plus amino acid sequences (B:S-A). However, this supergroup is poorly supported or not monophyletic in the three other analyses (Fig. 1). This is consistent with the fact that 'Amoebozoa' is defined largely by molecular phylogenies and lacks any clearly defined ultrastructural synapomorphies. In addition, our analyses fail to provide support for the 'unikont hypothesis', which argues for the monophyly of the 'Amoebozoa' plus 'Opisthokonta' [12,15,16,56-58]. The lack of support for 'unikonts' may reflect insufficient phylogenetic signal in our data sets. Alternatively, the hypothesized 'unikont' monophyly may be an artifact of limited taxon sampling in previous multigene studies.

The 'Rhizaria' receives only limited support (e.g., bootstrap support under RAxML <50% with the nucleotide data [R:n] and 6% under PHYML; Fig. 1). The 'Rhizaria' are supported by some published molecular phylogenies [42,59,60], but not by others [14]. The core Cercozoa show a sister-group relationship to the stramenopiles in all multigene analysis (Fig 2, 3, S1-S8), but without significant posterior probability or bootstrap support. This result is consistent with a recent multigene (85-protein) phylogenetic study from a limited number of taxa [30] that supports the sister relationship of *Reticulomyxa *(Foraminifera) plus *Bigelowiella *(chlorarachniophyte) with stramenopiles [41]. The relationship between 'Rhizaria' and Stramenopiles suggested by Hackett et al. (2007) has strong bootstrap and Bayesian support in their analyses and is the significantly favored topology using the approximately unbiased (AU) test. An independent study using a larger data set of nearly 30,000 amino acid positions also reported a specific relationship between 'Rhizaria' and 'Chromalveolates' [61]. This intriguing result needs to be tested using additional analyses that include more extensive taxon sampling.

The remaining three putative supergroups – 'Chromalveolata', 'Plantae', and 'Excavata' – are not found to be monophyletic (Fig. 1). In these analyses as in many others, members of the putative 'Excavata' are non-monophyletic [14]. This putative supergroup contains lineages whose ancestor is postulated to have had a distinctive feeding groove [1,24,62]. Here, we find two subclades of 'Excavata', albeit with mixed support (Fig. 1). The first group is consistent with the hypothesized 'Fornicata' [17,58]: Diplomonadida plus *Carpediemonas*. The second includes the Heterolobosea plus Euglenozoa, which share 'discoidal' mitochondria cristae and have been recovered in other multigene phylogenies [12,14]. The phylogenetic position of the putative 'Excavata' lineage *Malawimonas *is unstable in our analyses (Figs. 2, 3, see Additional files 2, 3, 4, 5, 6, 7, 8, 9) and more data are needed to test its relationship to other excavates.

The supergroup 'Plantae' – Rhodophyta (red algae), Glaucophyta, and Viridiplantae (green algae and land plants) – is consistently polyphyletic (Fig. 2, 3). The case for 'Plantae' monophyly is largely based on plastid encoded genes [63,64], plus recent evidence from some nuclear encoded proteins that are plastid targeted [65,66] and nuclear genes that encode cytosolic proteins [30,57,67]. Other lines of evidence for 'Plantae' monophyly come from analysis of the plastid machinery including plastid targeted metabolite translocator genes [68] and the shared protein import system embedded in the organelle membranes of 'Plantae' members (Tic-Toc system, [69]). Therefore, supergroup 'Plantae' may be monophyletic even though our present analysis lacks resolution with regard to this group. The 'Plantae' however remains controversial because its monophyly is not supported by several other multigene data sets using nuclear loci, thus retaining the possibility that this supergroup may be paraphyletic or polyphyletic [70-72].

We find no support for the putative supergroup 'Chromalveolata', despite the addition of numerous species from this lineage; i.e., 43 putative members in the 105 taxon dataset. The chromalveolate hypothesis unites the chlorophyll *c*-containing photosynthetic eukaryotes and their relatives and includes the cryptophytes, haptophytes, stramenopiles, apicomplexa, dinoflagellates, and ciliates [20]. The common origin of the plastid in chromalveolates, like in the Plantae, is supported by plastid multigene analyses [63,64,73], trees inferred from plastid-targeted proteins such as GAPDH and FBA [74-76] and plastid translocator genes (for apicomplexans, haptophytes, and stramenopiles, [68]).

Relationships among 'Chromalveolata' were recently tested using a 16-nuclear protein dataset that provided moderate bootstrap support for 'Chromalveolata' monophyly when including 'Rhizaria' (see inset in Fig. 1; [30]). However, most nuclear (host) trees using single and multigene analyses provide limited or no support for the monophyly of this supergroup (reviewed in [14]). Most clearly, our trees as well as recent published studies [30] refute the 'Chromista' hypothesis because we find no support for the monophyly of haptophytes plus stramenopiles plus cryptophytes, as is found in some plastid gene trees [54]. Instead, our 92 taxon tree (Fig. 3) supports the monophyly of stramenopiles and Alveolata (ciliates, apicomplexa, and dinoflagellates) that is consistent with the results of other studies [12,30]. Given that 'Chromista' is invalid then the 'Chromalveolata' hypothesis as proposed by Cavalier-Smith [20] is also falsified by our study.

### Impact of Taxon Sampling

We assessed how the removal of known problematic taxa affected the support for clades with ultrastructural identities and for putative supergroups using the reduced 92-taxon dataset (Figs. 1, 3, see Additional files 6, 7, 8, 9). Although we see an increase in support for clades with ultrastructural identities, the reduced taxon dataset shows little improvement for most supergroups. For example, there is an increase in support for groups such as the Heterolobosea (i.e., posterior probability support increases from 0.77 to full support under a B-n analysis; Fig. 1) and red algae (i.e., posterior probability support increases from 0.69 to 0.85 in B-n analysis; Fig 1.) There is also an increase in support for two supergroups, 'Amoebozoa' and 'Opisthokonta,' in our 92-taxon analysis (Fig. 1). For example, posterior probability support for the 'Amoebozoa' increases from <0.50 to 0.85 in the B-n analysis. A result of removing the long-branch Foraminifera to generate our 92 taxon set is that we no longer can assess the 'Rhizaria' because the remaining members represent only the subclade 'Cercozoa', as indicated in Figure 1; this subclade does show robust support with the 92 taxon data set (Fig. 1). Removal of problematic taxa does not provide any support for three supergroups, 'Chromalveolata,' 'Excavata,' and 'Plantae.'

Explanations for the limited support or lack of monophyly of the supergroups (with the exception of 'Opisthokonta' and to a lesser extent 'Amoebozoa') include: (1) taxon and gene sampling is too limited to support these deep relationships and (2) these putative supergroups do not reflect accurately deep relationships within eukaryotes. Disentangling these alternatives will require the use of both a broad taxon sampling, as used here, combined with greater sequence data.

## Conclusion

Intriguingly, the level of support in these analyses of four genes, including numerous newly-characterized sequences, matches what emerged from a review of the literature on molecular phylogenetic analyses of eukaryotes in general [14]. In both the analyses presented here and in our synthesis of the literature [14] the 'Opisthokonta' receive relatively strong support, the 'Amoebozoa' receive low to moderate support, and the remaining four supergroups ('Excavata', 'Rhizaria', 'Plantae' and 'Chromalveolata') are unsupported. As discussed above, the core 'Cercozoa' within 'Rhizaria' do show a sister-group relationship to stramenopiles, though our trees provide only Bayesian support for this result (see Fig. 3). This association of some 'Rhizaria' with some members of the 'Chromalveolata' calls into question the taxonomic validity of these two supergroups.

As we are using the same set of genes that are present in many other analyses (including some of those used to establish the putative supergroups), there is some circularity in the comparison between our and previously published analyses. Hence, assessment of potential supergroups must await analyses of novel gene data sets sampled from many taxa, in particular including enigmatic taxa such as *Ancyromonas *and *Malawimonas *that could potentially form independent lineages. Ultimately, resolving deep nodes will require the use of multigene alignments incorporating a wide diversity of taxa combined with the identification of robust ultrastructural or molecular synapomorphies for proposed clades.

## Methods

### Cultures and molecular methods

One hundred and five species from all six eukaryotic supergroups were used in this study. We obtained cultures from the American Type Culture Collection (ATCC), the Provasoli-Guillard National Center for Culture of Marine Phytoplankton (CCMP) and the Culture Collection of Algae at the University of Texas at Austin (UTEX). Cells were frozen in liquid nitrogen and ground with glass beads using a glass rod and/or Mini-BeadBeater™ (Biospec Products, Inc., Bartlesville, OK, USA). Total genomic DNA was extracted using the DNeasy Plant Mini Kit (Qiagen, Santa Clarita, CA, USA). Some DNA samples were obtained directly from the American Type Culture Collection (ATCC).

Primers for SSU-rDNA genes are from Medlin *et al*. [77] with three additional primers that were used to generate overlapping sequences from each clone as described in Snoeyenbos-West *et al*. [78]. Other primers were designed for actin, alpha-tubulin and beta-tubulin from broad eukaryotic alignments of these genes. PCR amplification was carried out using the following primers: actin, AAC TGG GAY GAY ATG GAR AAG AT and ATC CAC ATY TGY TGG AAN GT; beta-tubulin, GGT GCT GGT AAY AAY TGR GC and ACC AGG TCG TTC ATR TTN GA; alpha tubulin, initial PCR with CTA GGC AAY GCN TGY TGG GA and CAT GCC TTC NCC NAC RTA CC reamplified with nested primers TTG TAC TGC YTN GAR CAY G and AC GTA CCA GTG NAC RAA NGC. Phusion DNA Polymerase, a strict proofreading enzyme, was used to amplify our genes of interest and we have used the Lucigen PCRSmart, Novagen Perfectly Blunt, Invitrogen Zero Blunt TOPO, Invitrogen TOPO TA cloning kits. Sequencing of cloned plasmid DNA was done using vector- or gene-specific primers and the BigDye™ terminator kit (PE-Applied Biosystems, Norwalk, CT, USA). Sequences were run on an ABI 3100 automated sequencer. We have fully sequenced 2–4 clones of each gene for each organism and surveyed up to 8 clones per taxon in order to detect potential paralogs.

### Data analysis

To align SSU-rDNA sequences, we used HMMER v2.1.4 [79] whereas protein-coding genes were aligned by Clustal W [80]. For the SSU-rDNA alignment, we aligned the sequences using HMMER while incorporating secondary structure. These sequences were downloaded from The European Ribosomal Database [81]. The resulting alignment was further edited manually in MacClade v4.05 [82]. Protein coding genes were aligned as amino acids using Clustal W [80] as implemented in DNAstar's Lasergene software and manually adjusted in MacClade v4.05 [82]. For the phylogenetic analysis, we restricted our analysis to unambiguously aligned regions for which we were confident in positional homology as assessed by eye. For a subset of our analyses, we tried two different masks (conservative vs. liberal) of ambiguous positions and found no significant differences in inferences from topologies and support (data not shown).

Genealogies were inferred using MrBayes [83], RAxML [84] and PHYML [85]. Bayesian analyses were performed with the parallel version of MrBayes 3.1.2 using the GTR+I+ Γ (for nucleotide) and RtREV (for amino acid) models of sequence evolution [86]. Four to 16 simultaneous MCMCMC chains were run for 4 million generations sampling every 100 generations. Stationarity in likelihood scores was determined by plotting the -1nL against the generation. All trees below the observed stationarity level were discarded, resulting in a 'burnin' that comprised 25% of the posterior distribution of trees. The 50% majority-rule consensus tree was determined to calculate the posterior probabilities for each node. RAxML was run for 100 iterations using GTRGAMMA model for nucleotide data and PROTGAMMA with matrix RtREV for amino acid data. The datasets were partitioned to allow RaxML to assign different parameters for each gene. One hundred replicates for bootstrap analyses were run in RAxML and PHYML, and a 50% majority rule consensus was calculated to determine the support values for each node. MrModelTest [87] and ProtTest 1.3 [88] were used to select the appropriate model of sequences evolution for the nucleotides and amino acid data, respectively.

## Authors' contributions

HSY: led on data collection and analysis, contributed to writing; JG: led on data collection and analysis, contributed to writing; YIT: contributed to data collection and analysis, contributed to writing; MW: contributed to data collection; BCC: contributed to data collection; JCC: contributed to data collection; JML: contributed to data collection; DJP: contributed to data interpretation and writing;

DB: oversaw data collection and analysis, contributed to writing and revision; LAK: oversaw project (data collection, data analysis) and led on much of the writing.

## Supplementary Material

Additional file 1 **Table 1**. Table of taxa sampled and sources of genes. Click here for file

Additional file 2 **Figure 4**. Likelihood analysis of the 105-taxon data set of SSU-rDNA + nucleotide sequences of actin, alpha-tubulin and beta-tubulin performed with RaxML. See text and Figure 2 for additional notes. Click here for file

Additional file 3 **Figure 5**. Bayesian analyses of the 105-taxon data set of SSU-rDNA + nucleotide sequences of actin, alpha-tubulin and beta-tubulin performed with MrBayes. See text and Figure 2 for additional notes. Click here for file

Additional file 4 **Figure 6**. Bayesian analyses of the 105-taxon data set of amino acid sequences of actin, alpha-tubulin and beta-tubulin performed with MrBayes. See text and Figure 2 for additional notes. Click here for file

Additional file 5 **Figure 7**. Likelihood analysis of the 105-taxon data set of amino acid sequences of actin, alpha-tubulin and beta-tubulin performed with PhyML. See text and Figure 2 for additional notes. Click here for file

Additional file 6 **Figure 8**. Likelihood analysis of the 92-taxon data set of SSU-rDNA + nucleotide sequences of actin, alpha-tubulin and beta-tubulin performed with RaxML. See text and Figure 2 for additional notes. Click here for file

Additional file 7 **Figure 9**. Bayesian analyses of the 92-taxon data set of SSU-rDNA + nucleotide sequences of actin, alpha-tubulin and beta-tubulin performed with MrBayes. See text and Figure 2 for additional notes. Click here for file

Additional file 8 **Figure 10**. Bayesian analyses of the 92-taxon data set of amino acid sequences of actin, alpha-tubulin and beta-tubulin performed with MrBayes. See text and Figure 2 for additional notes. Click here for file

Additional file 9 **Figure 11**. Likelihood analysis of the 92-taxon data set of amino acid sequences of actin, alpha-tubulin and beta-tubulin performed with PhyML. See text and Figure 2 for additional notes. Click here for file

Additional file 10 **Figure 12**. Bayesian analysis of 93 actin amino acid sequences that are in the 105 multigene taxon analysis, performed with MrBayes. See text and Figure 2 for additional notes. Click here for file

Additional file 11 **Figure 13**. Bayesian analysis of 96 alpha-tubulin amino acid sequences that are in the 105 multigene taxon analysis, performed with MrBayes. See text and Figure 2 for additional notes. Click here for file

Additional file 12 **Figure 14**. Bayesian analysis of 98 beta-tubulin amino acid sequences that are in the 105 multigene taxon analysis, performed with MrBayes. See text and Figure 2 for additional notes. Click here for file
