# Supplementary material for: Broadly sampled multigene trees of eukaryotes
Source: BMC Evol Biol. 2008 Jan 18;8:14. doi: 10.1186/1471-2148-8-14 (PMC2249577; doi:10.1186/1471-2148-8-14)
Supplement: Additional file 1 — Table 1. Table of taxa sampled and sources of genes. [file 1471-2148-8-14-S1.DOC]

Table 1: Taxa and genes used in the study with the gene sources (generally GenBank accession numbers) shown.

| **Taxon** | **SSU** | **actin** | **atub** | **btub** |
| --- | --- | --- | --- | --- |
| *Acrasis Rosea* | AF011458 | NA | U66905 | AF276945 |
| ^*Allogromia* sp*.* | X86093 | AJ132370 | NA | AY818726 |
| *Amphidinium* (composite) | AF274252 | U84289 | AF482406 | AF482405 |
| *Ancyromonas* sp*.* ATCC 50267 | EF455780^4^ | EF455782^1^ | EF455765^1^ | EF455766^1^ |
| *Apodachlya brachynema* | AJ238663 | AY729840 | AY729828 | AY729815 |
| *Arabidopsis thaliana* | AB182642 | NM112046 | NM100360 | NM101856 |
| *Bigelowiella natans* CCMP 621 | AF054832 | EF455788^4^ | EF455792^4^ | EF455778^4^ |
| ^*Caenorhabditis elegans* | AY284652 | X16797 | NM_060199 | NM_065405 |
| *Candida albicans* | M60302 | X16377 | U38534 | M19398 |
| *Carpediemonas membranifera* | AY117416 | NA | AY117421 | AY117422 |
| *Cercomonas* sp*.* ATCC PRA-21 | EF455759^2^ | EF455793^2^ | EF455787^2^ | EF455796^2^ |
| ^*Chilodonella uncinata* ATCC 50194 | AF300281 | ABG81339 | AY041128 | ABG81322 |
| *Chilomonas paramecium* SAG 977.2a | AJ420676 | EF455726^1^ | EF455733^1^ | EF455742^1^ |
| *Chlamydomonas reinhardtii* | M32703 | D50838 | M11447 | K03281 |
| *Chroomonas mesostigmatica* CCMP269 | AF508268 | EF455727^1^ | NA | EF455743^1^ |
| *Chrysochromulinda polylepis* CCMP1757 | AJ004866 | EF455728^1^ | EF455734^1^ | EF455744^1^ |
| *Crypthecodinium cohnii* | M64245 | AF421536 | NA | AF421537 |
| *Cryptosporidium parvum* | X64341 | AF382338 | AF082877 | U65381 |
| ^*Cyanidioschyzon merolae* | AB158485 | AB095179 | AB095180 | AB095181 |
| *Cyanidium caldarium* | AB090833 | NA | AY302129 | AY302130 |
| *Cyanophora paradoxa* | X81840 | U90325 | AF119172 | AF092952 |
| *Danio rerio* | BX537263 | NM131591 | NM194388 | NM198809 |
| *Diacronema (composite)* CCMP 610 | AF106056 | EF455729^1^ | EF455735^1^ | EF455745^1^ |
| *Dictyostelium discoideum* | X00134 | XM_641596 | L13999 | AF030823 |
| *Dimorpha* sp*.* ATCC PRA54 | EF455769^1^ | EF455772^1^ | EF455768^1^ | EF455774^1^ |
| *Dinophyceae* sp. | AY251288 | AF482423 | NA | AF482424 |
| *Diplomonad* sp. | U93083 | NA | U29440 | U29441 |
| *Drosophila melanogaster* | M21017 | AE014298 | AE014297 | AE013599 |
| *Eimeria tenella* | U40264 | Sang: Et_v1_TwnscnContig7015.tmp5 | Sang: Et_v1_Twnscn_ | Sang: Et_v1_Twnscn_ |
|  |  |  | Contig6762.tmp2 | Contig6045.tmp12 |
| ^*Encephalitozoon cuniculi* | L07255 | NC_003242 | NC003234 | NC003230 |
| ^*Entamoeba histolytica* | X56991 | M19871 | L07898 | XM_652078 |
| *Eremothecium gossypii* | AY046265 | NM208524 | NA | NM208980 |
| *Euglena gracilis* | AF283308 | AF057161 | AF182557 | AF182558 |
| ^*Euplotes octocarinatus* | AJ310489 | NA | X69466 | X69467 |
| *Galdieria sulphuraria* DBV074 JAVA | MSU:contig_633 | MSU:contig_1017 | MSU:contig_1530 | MSU:contig_1384 |
| *Gallus gallus* | DQ018752 | AF012348 | V00388 | M11442 |
| ^*Giardia lamblia* | M54878 | L29032 | XM_774477 | X06748 |
| *Glaucocystis nostochinearum* UTEXB1929 | X70803 | PEP GNL00000708 | EF455736^2^ | EF455746^2^ |
| *Goniomonas truncata* | U03072 | AF284837 | AF050087 | AF050089 |
| *Guillardia theta* | X57162 | AF284835 | AF050090 | AF050091 |
| *Heterocapsa rotundata* | AF274267 | AF482409 | AF482410 | NA |
| *Heterosigma akashiwo* | AB001287 | AY729842 | AY729829 | AY729817 |
| *Hexamita inflata* | L07836 | NA | U93088 | AY277792 |
| *Homo sapiens* | X03205 | NM001100 | NM032704 | NM001069 |
| *Hyperamoeba* sp*.* ATCC PRA39 | EF455771^2^ | EF455784^1^ | EF455783^1^ | EF455801^1^ |
| *Isochrysis galbana* CCMP1323 | IGA246266 | EF455764^3^ | AY729830 | EF455758^2^ |
| *Jakoba incarcerata* | AY117419 | NA | AF267179 | AF267183 |
| *Jakoba libera* ATCC50992 | AF411288 | EF455770^1^ | AF267180 | AF267184 |
| *Karenia brevis* CCMP718 | AF274259 | AF482415 | AF482419 | EF455751^2^ |
| *Leishmania major* | NC_007268 | XM883541 | SangLmjF13.0380 | X93566 |
| ^*Loxodes striatus* | U24248 | AB097686 | Z49852 | NA |
| *Malawimonas jakobiformis* ATCC 50310 | EF455761^4^ | EF455790^1^ | AF267181 | AF267185 |
| *Mallomonas rasilis* | U73231 | AY729844 | AY729831 | NA |
| *Mantoniella squamata* CCMP480 | X73999 | EF455730^1^ | EF455737^4^ | EF455747^4^ |
| *Mesostigma viride* CCMP2046 | AJ250108 | AAC16055 | EF455738^3^ | EF455748^2^ |
| ^*Metopus palaeformis* CCAP1653/3 | M86385 | EF455754^1^ | AY041136 | NA |
| *Micromonas pusilla* CCMP490 | AB183589 | NA | EF455739^1^ | EF455749^4^ |
| *Monocercomonas* sp. | U17507 | NA | U66903 | AY277791 |
| *Monosiga brevicollis* | AF100940 | AY026072 | AY026070 | AY026071 |
| *Mus musculus* | X00686 | NM007392 | NM009446 | NM009450 |
| *Naegleria gruberi* | M18732 | AF101729 | X81049 | X81050 |
| *Nitzschia thermalis* | AY485458 | AY713395 | AY713396 | NA |
| ^*Nyctotherus ovalis* from cockroach | AJ009701 | EF455753^2^ | AY041145 | NA |
| *Oryza sativa* | AF069218 | AB047313 | AF182523 | D13224 |
| *Oxyrrhis marina* | AB033717 | AF482402 | AF482403 | AF482404 |
| *Paramecium caudatum* | AF217655 | AB070223 | AB035413 | AB070222 |
| *Pavlova gyrans* CCMP607 | U40922 | EF455731^1^ | EF455740^1^ | NA |
| *Pavlova lutheri* | AJ515247 | PLL00000105 | AY729833 | AY729820 |
| *Pelagomonas calceolata* CCMP1214 | EF455763^2^ | EF455791^1^ | EF455800^1^ | EF455781^1^ |
| *Peridinium willei* | AF274272 | AF482420 | NA | AF482421 |
| *Perkinsus marinus* | AF126013 | U84287 | AF482399 | AF482400 |
| *Phaeodactylum tricornutum* | AJ269501 | AY729845 | AY729834 | AY729821 |
| *Physarum polycephalum* | X13160 | X07792 | X14213 | X12371 |
| *Physcomitrella patens* | AF126289 | AY382282 | AB096718 | AY382286 |
| *Phytophthora palmivora* | AY742745 | AY729846 | AY729835 | AY729822 |
| *Plasmodium berghei* | AJ243513 | XM_668904 | DQ070855 | M58750 |
| *Plectospira myriandra* | NA | AY729847 | AY729836 | AY729823 |
| *Polysphondylium violaceum* | AY040340 | NA | AF276946 | AF276947 |
| *Porphyra purpurea* | AF358405 | DQ111776 | NA | Z67991 |
| *Porphyra yezoensis* | D79976 | AB039831 | ABJ80981 | AY221630 |
| *Prorocentrum minimum* | AJ415520 | U84290 | NA | AF274884 |
| *Prymnesium parvum* | AJ246269 | AY729848 | AY729837 | AY729824 |
| *Reclinomonas americana* | AF053089 | NA | AF267182 | AF267187 |
| ^*Reticulomyxa filosa* | AJ132367 | AJ132375 | X96475 | X96478 |
| *Rhizopus microsporus* | AF157158 | AJ287197 | AY138787 | AF162064 |
| *Saccharomyces cerevisiae* | AY799853 | D50617 | NC001145 | NC001138 |
| *Schizosaccharomyces pombe* | AY046272 | Y00447 | NM_001023795 | NM_001022572 |
| *Stephanopogon apogon* ATCC50096 | NA | EF455762^2^ | EF455760^1^ | EF455757^1^ |
| *Storeatula* sp. CCMP1868 | AY702130 | EF455732^1^ | EF455741^1^ | EF455750^1^ |
| *Streblomastix strix* | AY188885 | PEP | AY188855 | AY188856 |
| *Strongylocentrotus purpuratus* | L28056 | NM214469 | XM_779124 | X07502 |
| ^*Stylonychia lemnae* | AF164124 | DQ108617 | X12365 | AF510208 |
| *Tetrahymena thermophila* | AF429899 | M13939 | M86723 | L01416 |
| *Thalassiosira pseudonana* | AF374481 | JGI: 33.149.1 | JGI: 18.27.1 | JGI: 12.73.1 |
| *Thalassiosira weissflogii* | AF374477 | AJ002018 | NA | AF276908 |
| *Thaumatomonas seravini* ATCC50636 | EF455776^4^ | EF455755^1^ | EF455794^1^ | EF455799^1^ |
| *Thecamoeba-*like ATCC PRA35 | EF455775^1^ | EF455773^1^ | EF455756^1^ | EF455789^1^ |
| *Thraustotheca clavata* | NA | AY729851 | AY729839 | AY729827 |
| *Toxoplasma gondii* | U03070 | U10429 | M20024 | M20025 |
| *Trichomonas vaginalis* | U17510 | U63122 | AF327848 | L05468 |
| *Trichonympha agilis* | AB003920 | NA | AF230348 | AB107788 |
| *Trypanosoma brucei* | AL92960 | M20310 | XM_841656 | XM_841653 |
| *Ustilago maydis* | AY702258 | XM_757271 | XM_752275 | XM_756882 |
| *Volvox carteri* | X53904 | M33963 | X12846 | X12855 |
| *Zea mays* | AF168884 | AY273142 | X15704 | L10633 |

Notes: Taxa marked with ^ removed for 92 taxon dataset. Numbers in columns 2-5 are GenBank accession numbers except MSU = Michigan State University <http://genomics.msu.edu/galdieria/>; PEP = http://www.tagc.ca/pep.php ; Sang = Sanger sequencing center http://www.sanger.ac.uk/Projects/Protozoa/; JGI = Joint Genome Institute = http://genome.jgi-psf.org/. NA = not available. Two 'composite' entries were generated for concatenated analyses by combining genes from multiple species in a genus. Sequences added to GenBank in this study are marked as follows: ^1^ Newly characterized. ^2^ Previously characterized from same species but from different or unknown strain. ^3^ Newly characterized genomic sequences but also characterized as publically-available EST. ^4^ Previously characterized from same strain and confirmed here. Paralogs characterized in this study but not included in the analyses: *Bigellowiella natans* CCMP 621 beta-tubulin EF455767^4^; *Dimorpha* sp*.* ATCC PRA-54 actin EF455798^1^; *Metopus palaeformis* CCAP 1653/3 actins, EF455795^1^ and EF455754^1^; *Nyctotherus ovalis* actin, EF455797^1^**.**
