# Supplementary figures and images for: Broadly sampled multigene trees of eukaryotes
Source: BMC Evol Biol. 2008 Jan 18;8:14. doi: 10.1186/1471-2148-8-14 (PMC2249577; doi:10.1186/1471-2148-8-14)

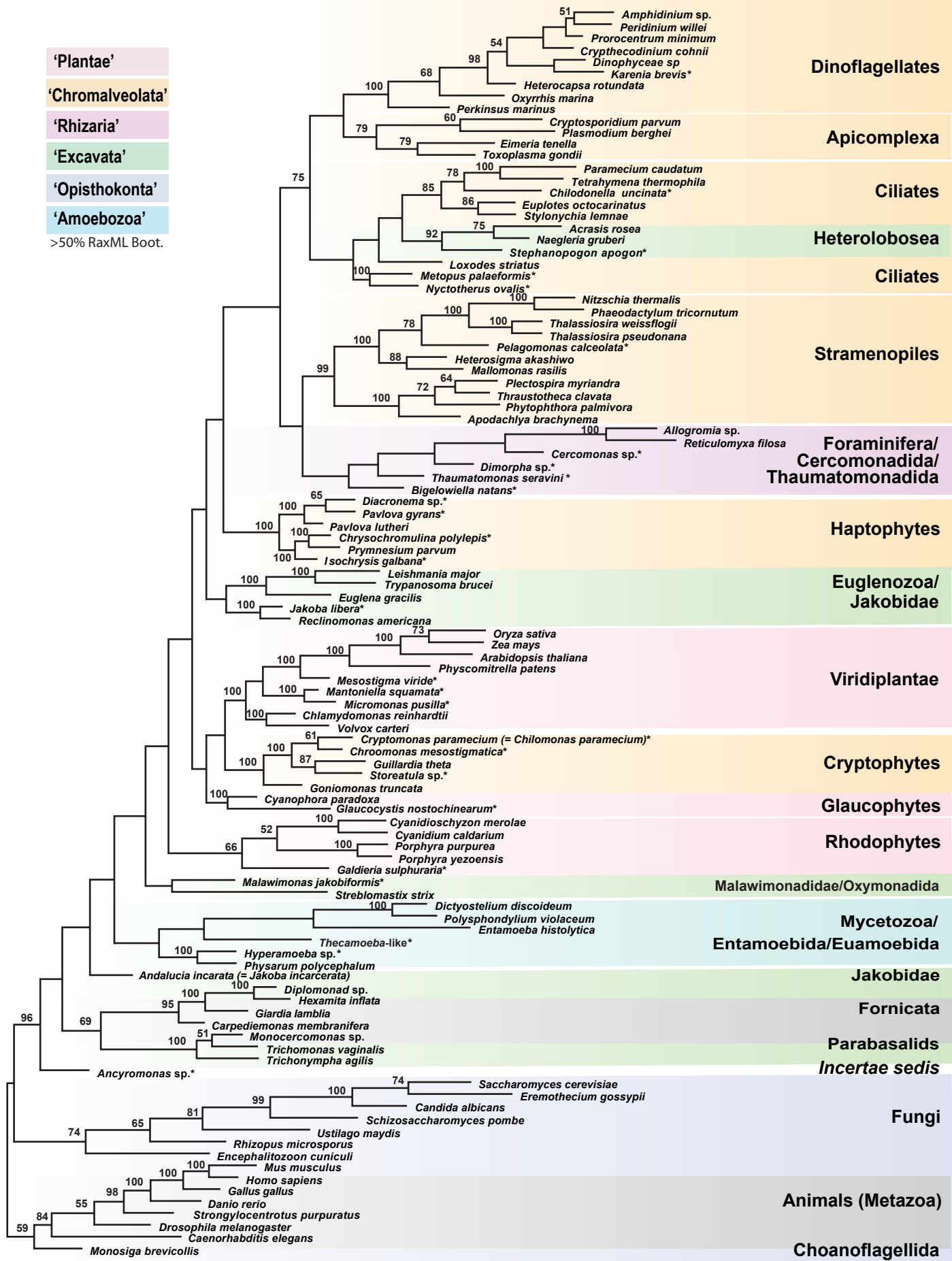

Supplement: Additional file 2 — Figure 4. Likelihood analysis of the 105-taxon data set of SSU-rDNA + nucleotide sequences of actin, alpha-tubulin and beta-tubulin performed with RaxML. See text and Figure 2 for additional notes. [file 1471-2148-8-14-S2.PDF]

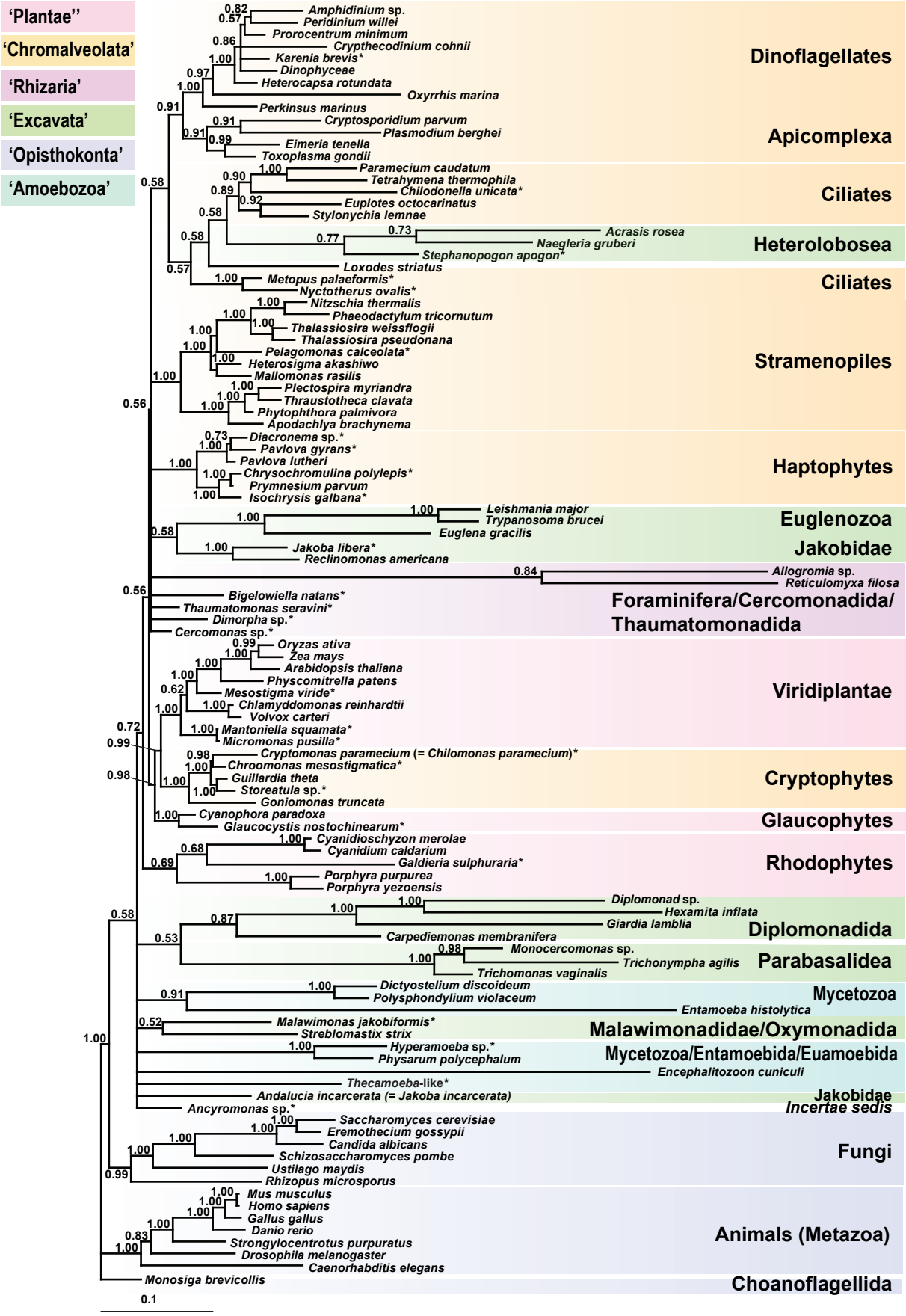

Supplement: Additional file 3 — Figure 5. Bayesian analyses of the 105-taxon data set of SSU-rDNA + nucleotide sequences of actin, alpha-tubulin and beta-tubulin performed with MrBayes. See text and Figure 2 for additional notes. [file 1471-2148-8-14-S3.PDF]

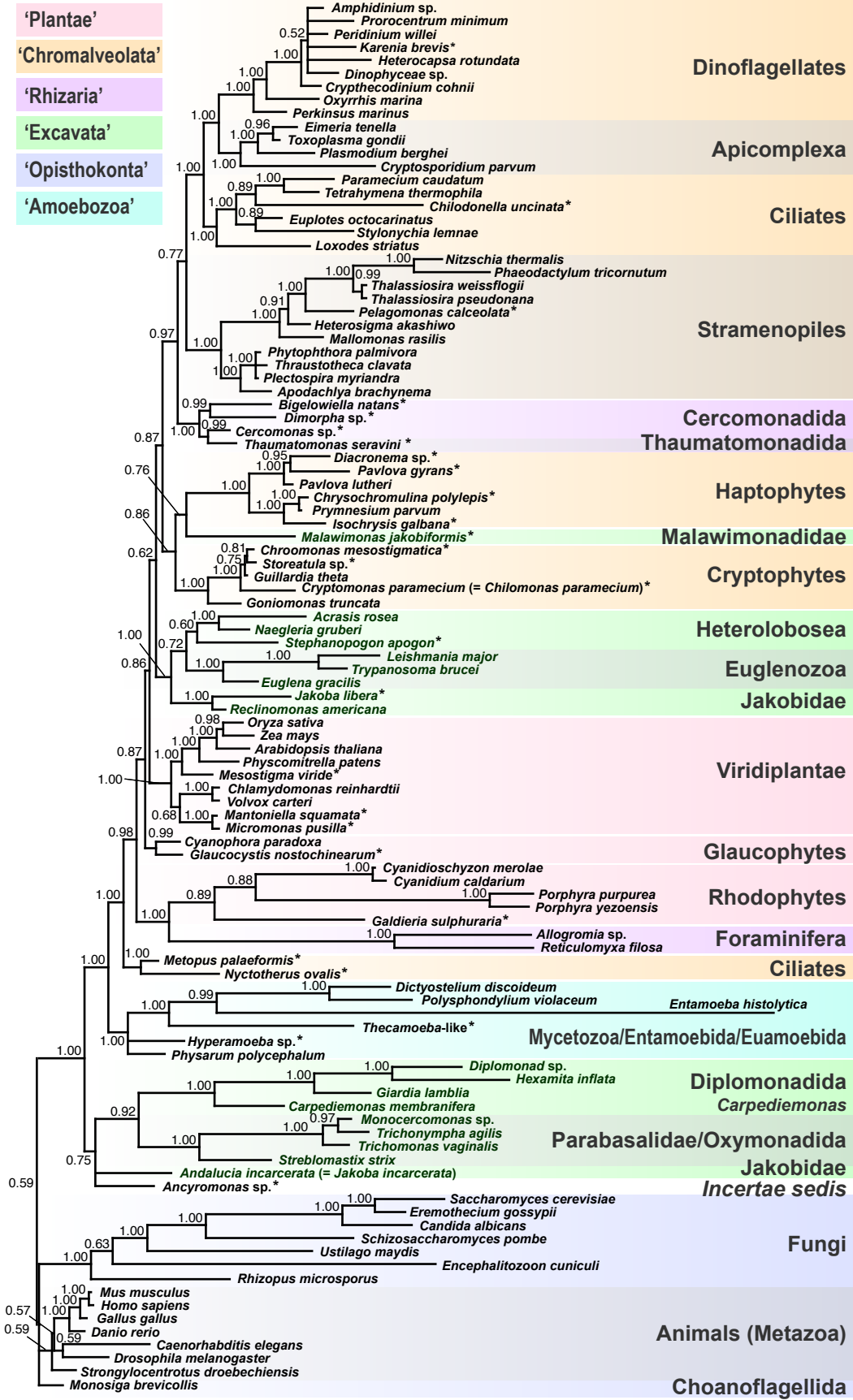

0.1 substitutions/site

Supplement: Additional file 4 — Figure 6. Bayesian analyses of the 105-taxon data set of amino acid sequences of actin, alpha-tubulin and beta-tubulin performed with MrBayes. See text and Figure 2 for additional notes. [file 1471-2148-8-14-S4.PDF]

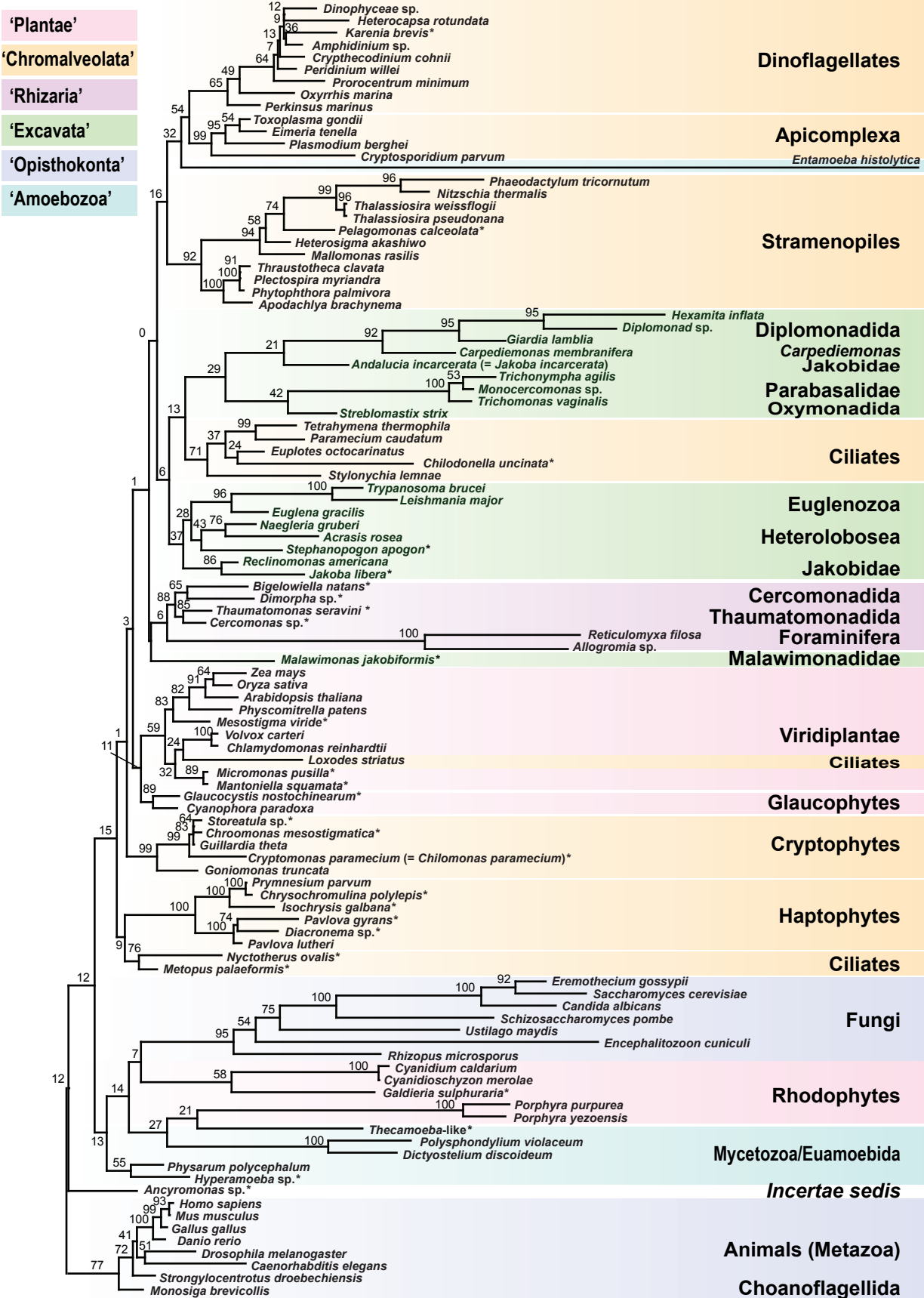

Supplement: Additional file 5 — Figure 7. Likelihood analysis of the 105-taxon data set of amino acid sequences of actin, alpha-tubulin and beta-tubulin performed with PhyML. See text and Figure 2 for additional notes. [file 1471-2148-8-14-S5.PDF]

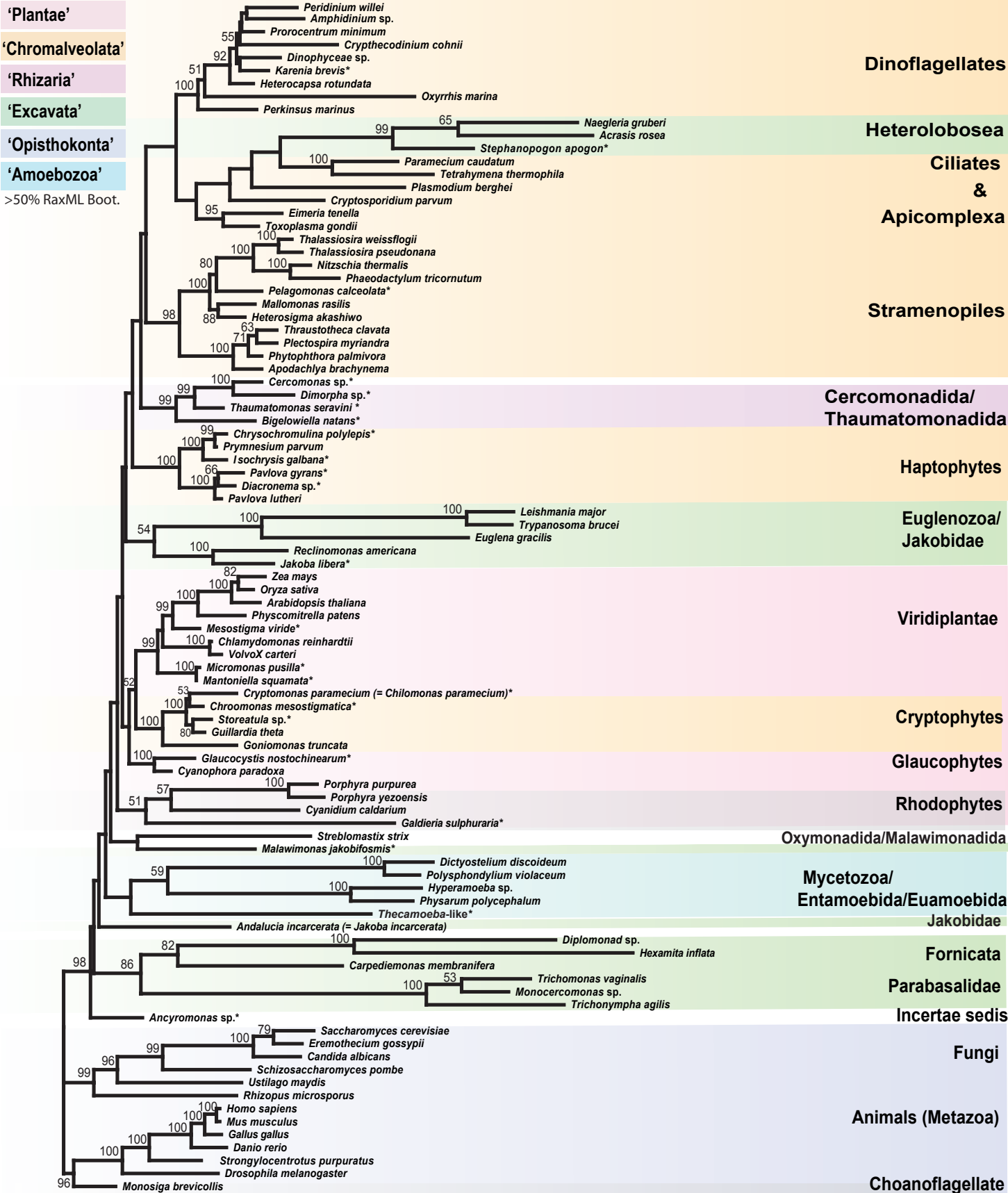

Supplement: Additional file 6 — Figure 8. Likelihood analysis of the 92-taxon data set of SSU-rDNA + nucleotide sequences of actin, alpha-tubulin and beta-tubulin performed with RaxML. See text and Figure 2 for additional notes. [file 1471-2148-8-14-S6.PDF]

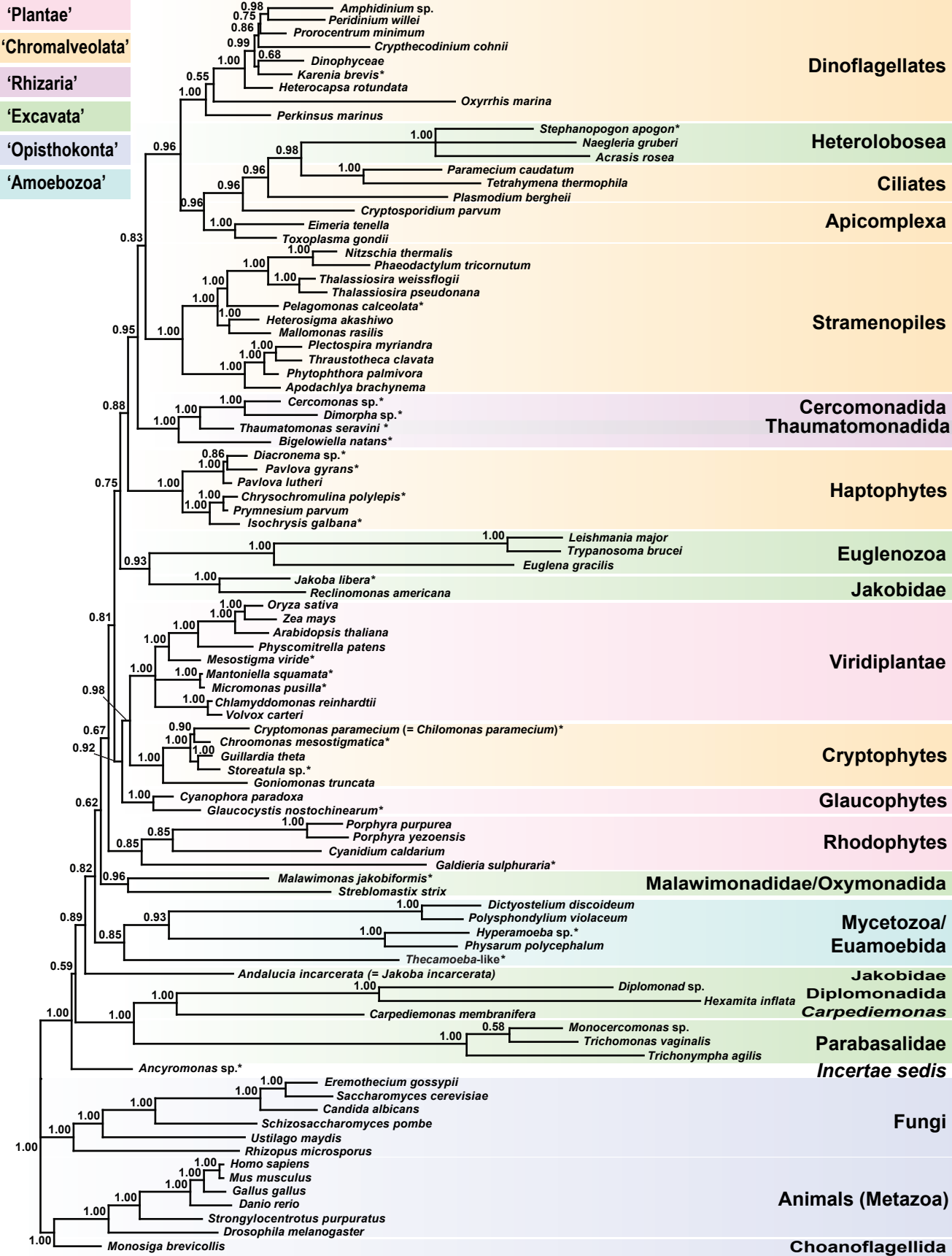

Supplement: Additional file 7 — Figure 9. Bayesian analyses of the 92-taxon data set of SSU-rDNA + nucleotide sequences of actin, alpha-tubulin and beta-tubulin performed with MrBayes. See text and Figure 2 for additional notes. [file 1471-2148-8-14-S7.PDF]

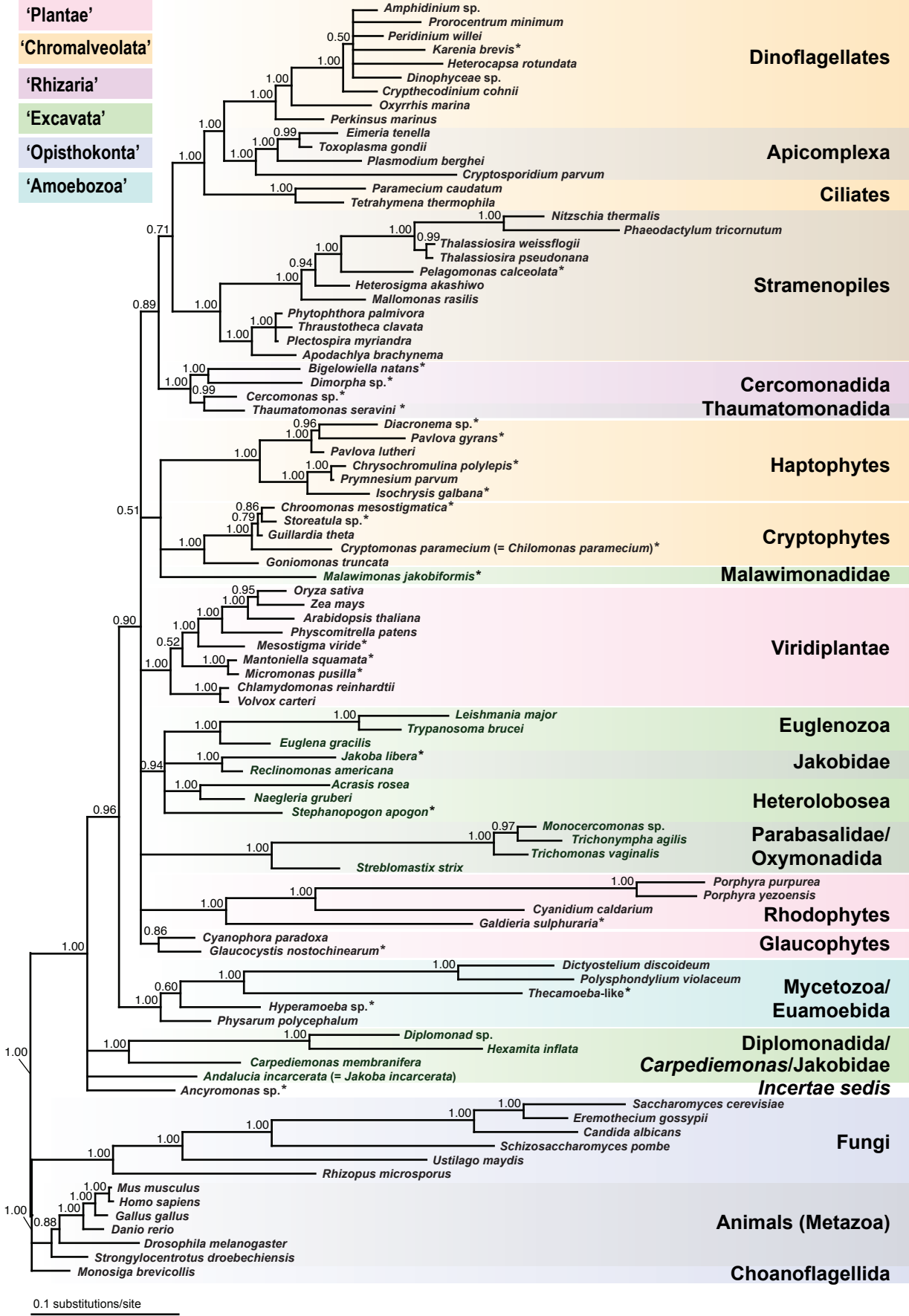

Supplement: Additional file 8 — Figure 10. Bayesian analyses of the 92-taxon data set of amino acid sequences of actin, alpha-tubulin and beta-tubulin performed with MrBayes. See text and Figure 2 for additional notes. [file 1471-2148-8-14-S8.PDF]

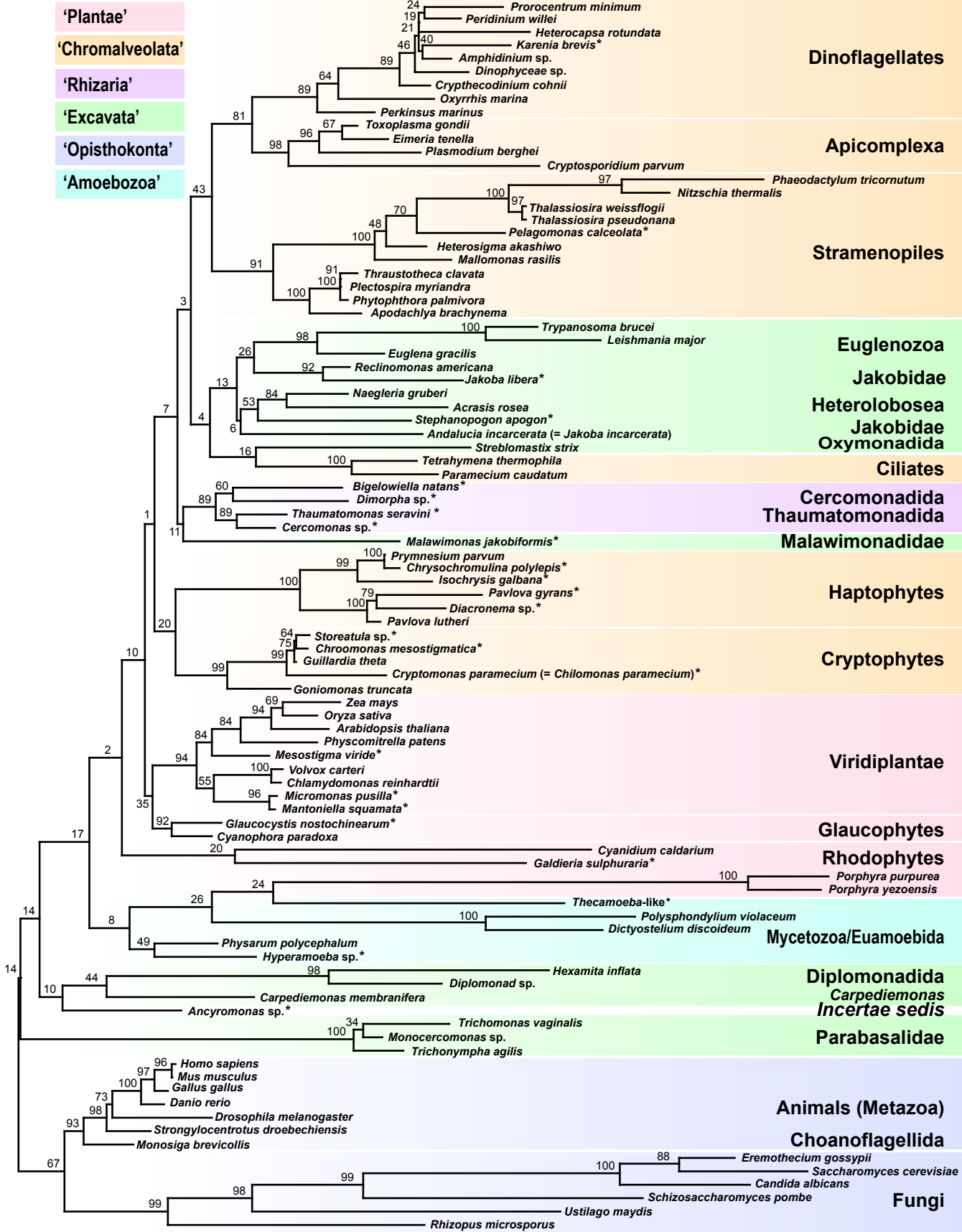

Supplement: Additional file 9 — Figure 11. Likelihood analysis of the 92-taxon data set of amino acid sequences of actin, alpha-tubulin and beta-tubulin performed with PhyML. See text and Figure 2 for additional notes. [file 1471-2148-8-14-S9.PDF]

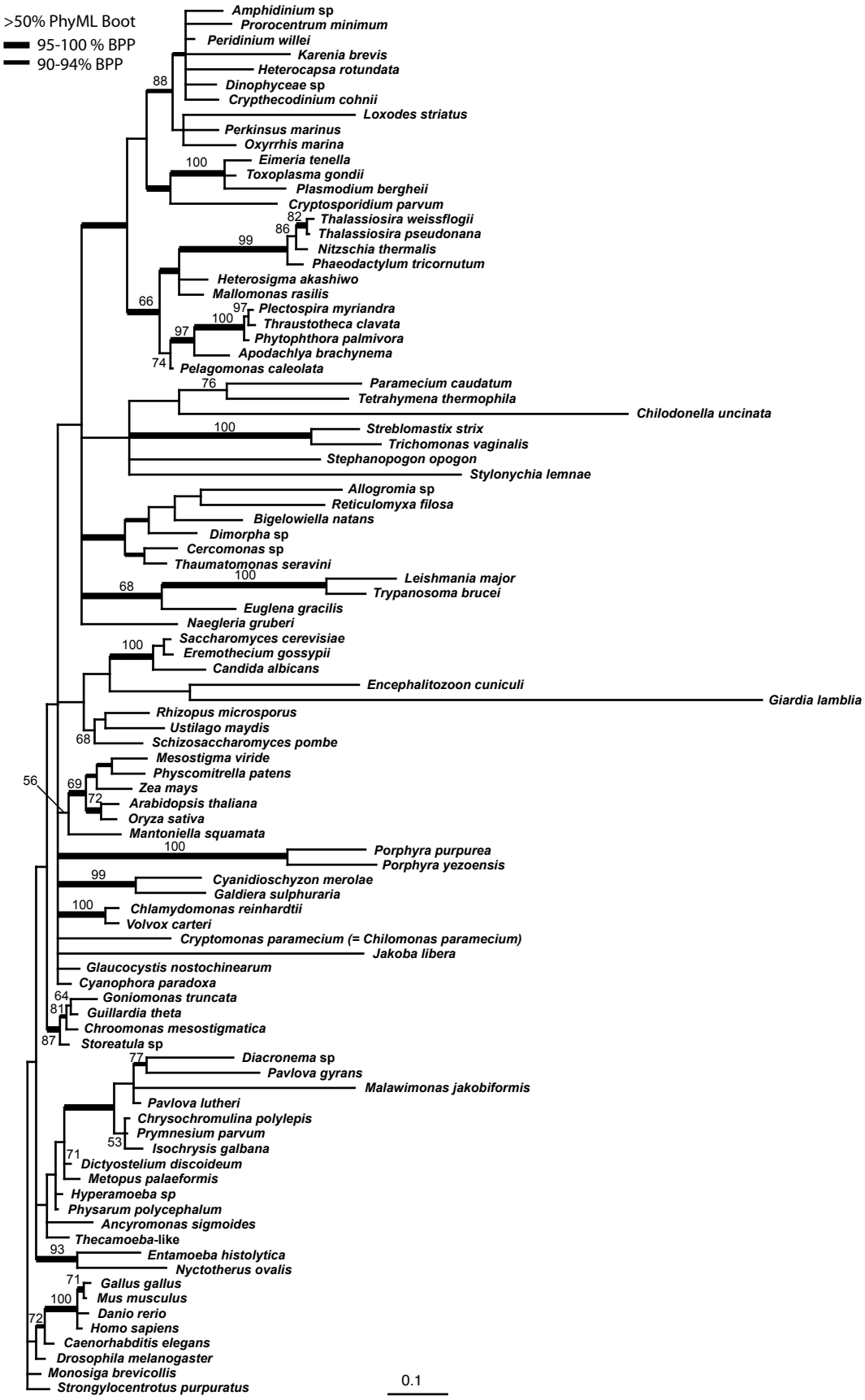

Supplement: Additional file 10 — Figure 12. Bayesian analysis of 93 actin amino acid sequences that are in the 105 multigene taxon analysis, performed with MrBayes. See text and Figure 2 for additional notes. [file 1471-2148-8-14-S10.PDF]

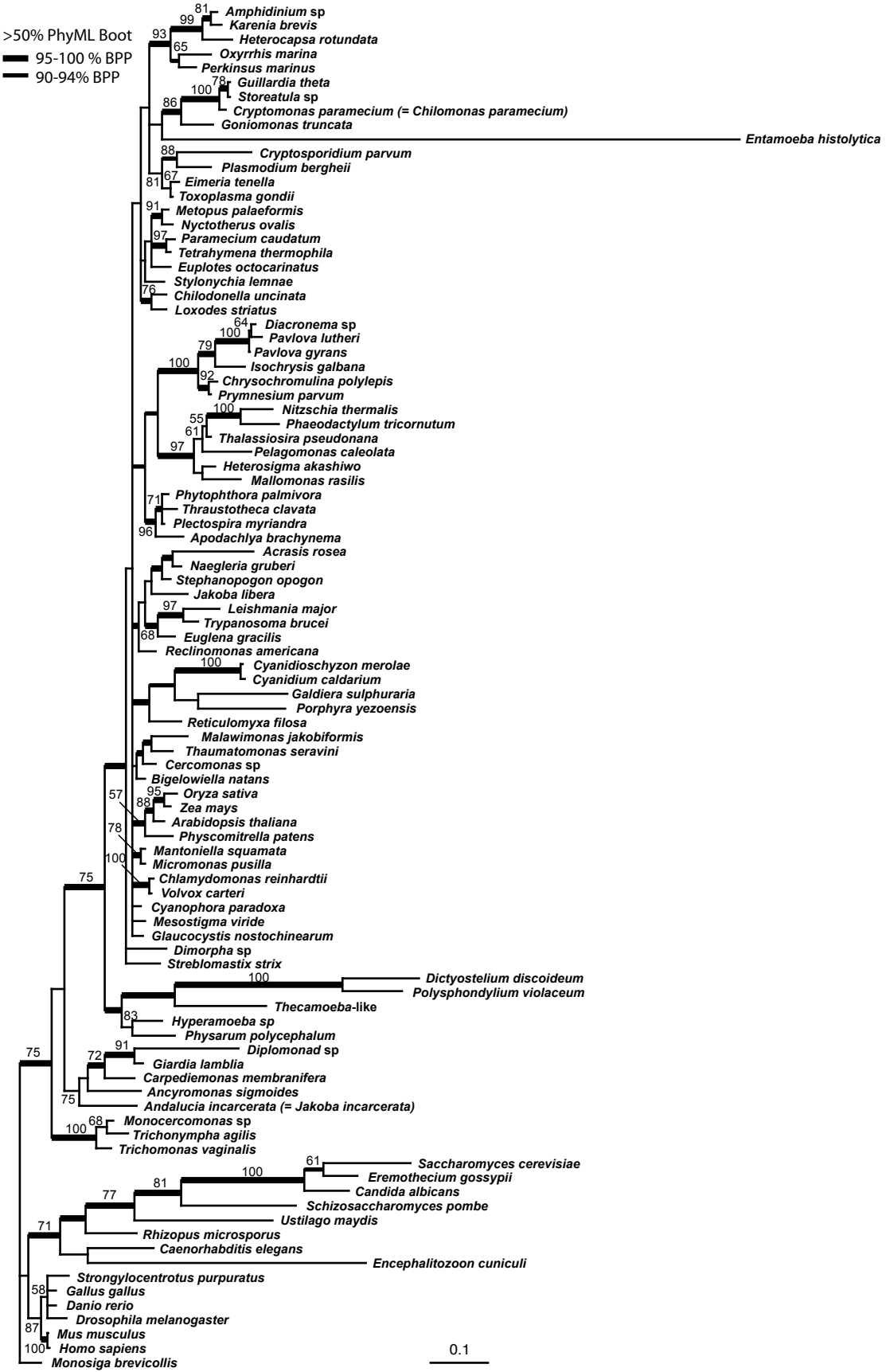

Supplement: Additional file 11 — Figure 13. Bayesian analysis of 96 alpha-tubulin amino acid sequences that are in the 105 multigene taxon analysis, performed with MrBayes. See text and Figure 2 for additional notes. [file 1471-2148-8-14-S11.PDF]

>50% PhyML Boot

95-100 % BPP

90-94% BPP

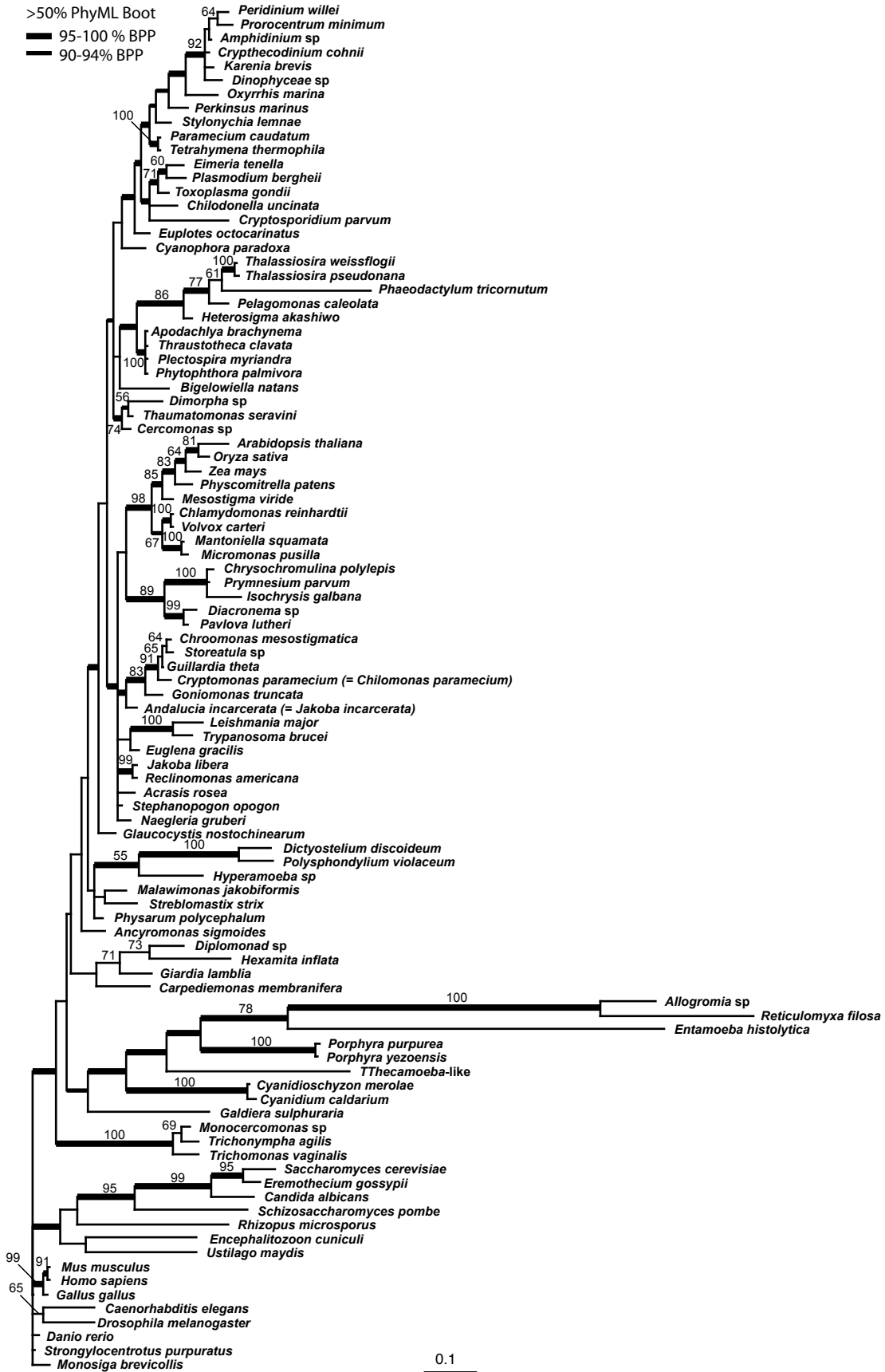

Supplement: Additional file 12 — Figure 14. Bayesian analysis of 98 beta-tubulin amino acid sequences that are in the 105 multigene taxon analysis, performed with MrBayes. See text and Figure 2 for additional notes. [file 1471-2148-8-14-S12.PDF]
